# Temporal Trends and Demographic Disparities in Respiratory Failure Mortality Among Adults with Chronic Liver Disease: A National Mortality Database Analysis, 1999 to 2024

**DOI:** 10.3390/diseases14070241

**Published:** 2026-07-03

**Authors:** Shubhendu Bajpai, Abdullah Sultany, Muhammad Sarmad Aleem, Sahil Grover, Ashraf Ullah, Eshal Amir, Kevin Carroll, Rahul Zain, Rewanth Katamreddy, Dushyant Singh Dahiya, Michelle Bernshteyn, Adam Breslin

**Affiliations:** 1 Department of Internal Medicine, Guthrie Robert Packer Hospital, Sayre, PA 18840, USA; 2 MBBS Program, Shadman Campus, College of Medicine, FMH, Lahore 54000, Pakistan; eshalamir05@gmail.com; 3 Department of Pulmonary and Critical Care Medicine, Guthrie Robert Packer Hospital, Sayre, PA 18840, USA; 4 Department of Gastroenterology and Hepatology, Guthrie Robert Packer Hospital, Sayre, PA 18840, USA; 5 Division of Gastroenterology, Hepatology and Motility, School of Medicine, University of Kansas, Kansas City, KS 66160, USA

**Keywords:** chronic liver disease, respiratory failure, mortality, joinpoint regression, health disparities, COVID-19

## Abstract

**Background:** Respiratory failure (RF) is a frequently fatal complication of chronic liver disease (CLD), yet population-level data on RF-related mortality trends among adults with CLD are lacking. This study characterized temporal trends and demographic disparities in RF-related mortality among U.S. adults with CLD from 1999 to 2024. **Methods:** Death certificate data were obtained from the CDC WONDER database for adults aged ≥25 years with both RF (ICD-10: J96) and CLD (ICD-10: K70–K76) listed as an underlying or contributing cause of death. Age-adjusted mortality rates (AAMRs) per 100,000 were calculated using the 2000 U.S. standard population. Joinpoint regression identified temporal inflection points and annual percentage change (APC). **Results:** Among 241,075 deaths, the overall AAMR increased 3.2-fold from 2.237 (1999) to 7.162 (2021) per 100,000, then declined to 6.132 by 2024. Joinpoint analysis identified four segments: moderate increase (1999–2006; APC +2.40%), accelerated increase (2006–2018; APC +5.37%), late acceleration period (2018–2021; APC +13.10%), and post-pandemic decline (2021–2024; APC −4.32%; all *p* < 0.001). The 2024 AAMR remained 174.2% above baseline. The male-to-female rate ratio narrowed from 2.02 to 1.50, with females showing steeper acceleration (+14.38% vs. +12.36%). American Indian or Alaska Native individuals had the highest AAMRs and the most dramatic surge (APC +26.90%). Rural areas surpassed urban AAMRs by 2020, with steeper post-2007 acceleration (+8.74% vs. +5.51%). The Western U.S. consistently had the highest regional rates. Younger adults aged 25–34 and 35–44 showed 2.96-fold and 2.37-fold increases in crude mortality rates, respectively. Approximately 80% of deaths occurred in inpatient settings. **Conclusions:** RF-related mortality among U.S. adults with CLD increased more than threefold from 1999 to 2021, with a dramatic surge followed by incomplete decline. Persistent disparities by sex, race/ethnicity, urbanization, and region highlight the need for targeted interventions, including expanded screening for alcohol-associated and metabolic liver disease and improved access to hepatology services in underserved communities.

## 1. Introduction

Chronic liver disease (CLD) constitutes a significant and escalating public health challenge in the United States [1,2,3]. In the past two decades, annual cirrhosis-related deaths have risen by over 65%, primarily due to increasing rates of alcohol-associated liver disease (ALD) and nonalcoholic fatty liver disease (NAFLD), now referred to as metabolic dysfunction-associated steatotic liver disease (MASLD) [1,2,3,4]. In contrast, mortality from hepatitis C virus (HCV)-related liver disease has declined following the introduction of direct-acting antiviral therapies [2,4]. These etiologic changes have altered the demographic and geographic distribution of CLD mortality, with disproportionate increases among younger adults, women, White Americans, American Indian or Alaska Native (AIAN) populations, and individuals residing in rural or socioeconomically disadvantaged areas [3,5,6,7]. The COVID-19 pandemic further accelerated CLD-related mortality, with cirrhosis-specific death rates rising by more than 11% annually from 2019 to 2021, largely due to increases in ALD- and NAFLD-related deaths [8,9,10]. Increases in alcohol consumption, reduced healthcare access, and social isolation during the pandemic compounded the pre-existing upward trend in liver disease burden [11,12,13].

Individuals with cirrhosis are at risk for pulmonary complications through two distinct pathophysiologic pathways [14,15]. First, CLD-specific pulmonary syndromes arise directly from portal hypertension and hepatic dysfunction. These include hepatopulmonary syndrome (HPS), characterized by intrapulmonary vascular dilatation and ventilation-perfusion mismatch affecting 5% to 30% of patients with cirrhosis and associated with a twofold increase in mortality risk [16,17,18]; portopulmonary hypertension (POPH), present in 5% to 10% of liver transplant candidates and characterized by elevated pulmonary vascular resistance that may result in right ventricular failure [16,17]; and hepatic hydrothorax, which can cause restrictive physiology and ventilatory insufficiency [19]. Second, individuals with CLD are susceptible to secondary respiratory complications associated with systemic critical illness, including acute respiratory distress syndrome (ARDS), pneumonia, and sepsis-related respiratory compromise, driven by cirrhosis-associated immune dysfunction, systemic inflammation, sarcopenia and diaphragmatic weakness, tense ascites compromising chest wall compliance, and metabolic syndrome-related restrictive lung disease [20,21]. The COVID-19 pandemic introduced an additional respiratory risk, as SARS-CoV-2 infection in patients with cirrhosis was associated with a 32% mortality rate, with respiratory failure accounting for 71% of deaths [22,23,24]. Critically ill patients with cirrhosis who develop acute-on-chronic liver failure (ACLF) often require mechanical ventilation, and respiratory organ failure is a major determinant of short-term mortality in this group [20]. Both CLD-specific and secondary pulmonary complications converge on respiratory failure as a final common pathway.

Respiratory failure was selected as the primary focus of this analysis because it represents the leading organ failure driving short-term mortality in critically ill patients with cirrhosis and acute-on-chronic liver failure [20]. In the largest international registry study of SARS-CoV-2 infection in patients with chronic liver disease, respiratory failure accounted for 71% of deaths [22]. Although prior Centers for Disease Control and Prevention Wide-Ranging Online Data for Epidemiologic Research (CDC WONDER) and National Vital Statistics System (NVSS) studies have comprehensively characterized overall CLD mortality trends, none have specifically examined respiratory failure as a contributing cause of death in this population [1,3,4,5,8]. Understanding the population-level burden of respiratory failure-related mortality among adults with CLD is therefore critical for identifying high-risk groups, allocating resources, and guiding targeted public health interventions [7,25]. The present study sought to characterize trends in respiratory failure-related mortality among adults with CLD in the United States from 1999 to 2024, stratified by sex, race and ethnicity, urbanization, geographic region, age, and place of death, using joinpoint regression analysis to identify statistically significant temporal inflection points.

## 2. Methodology

### 2.1. Study Setting and Population

In this retrospective cohort from 1999 to 2024, death certificate data was retrieved from CDC WONDER (Centers for Disease Control and Prevention Wide-Ranging Online Data for Epidemiologic Research) database and analyzed for Respiratory Failure (RF) mortality among adults with Chronic Liver Disease (CLD) aged ≥25 years [26,27]. ICD-10 codes (International Statistical Classification of Diseases and Related Health Problems, 10th Revision) were used to search the multiple cause of death data to identify causes: J96 for Respiratory Failure and K70–K76 for Chronic Liver Disease, respectively.

Deaths were identified using the CDC WONDER multiple cause of death files, which capture all conditions listed on the death certificate. Both RF (ICD-10: J96) and CLD (ICD-10: K70–K76) were required to appear anywhere on the death certificate, either as the underlying cause of death or as a contributing cause of death. This “any-mention” approach captures the full spectrum of RF-CLD co-occurrence on death certificates and is the standard methodology in multiple-cause-of-death research, consistent with prior CDC WONDER and NVSS studies of CLD mortality [1,3,8,9,28,29]. By requiring both conditions to appear on the same death certificate, the analysis identifies deaths in which RF and CLD were judged by the certifying physician to be clinically relevant to the death event. This approach is purely descriptive and does not assume a temporal or causal relationship between the two conditions; rather, it captures deaths in which both conditions were considered sufficiently important to be recorded on the death certificate.

This dataset includes cause of death information across all 50 states and the District of Columbia. The analysis was restricted to adults aged ≥25 years to focus on the population in which CLD is epidemiologically prevalent and to exclude pediatric and young adult deaths, which have distinct etiologies (e.g., biliary atresia, congenital hepatic fibrosis, metabolic liver diseases). This age threshold is consistent with prior CDC WONDER studies of CLD and hepatocellular carcinoma mortality [3,5,30,31]. Our study was exempted from local institutional review board (IRB) approval as the data used was de-identified and publicly accessible. This was done in accordance with the STROBE (Strengthening the Reporting of Observational Studies in Epidemiology) guidelines [32].

### 2.2. Data Extraction

Data extraction was performed based on population size, year and location of death, demographics, urban–rural classification, and census region. Place of death included medical facilities (outpatient, emergency room, inpatient, dead on arrival, or status unknown), home, hospice, and nursing home/long-term care facilities. Race/ethnicity was categorized as Hispanic or Latino and Non-Hispanic; non-Hispanic categories include American Indian or Alaska Native, Asian or Pacific Islander, NH White, and NH Black or African American. Urban–rural status was determined using the National Center for Health Statistics Urban-Rural Classification Scheme, which defines counties as urban (large metropolitan area [population ≥ 1 million], medium/small metropolitan area [population 50,000–999,999]) or rural (population < 50,000), according to the 2013 U.S. Census classification [33]. Additionally, U.S. regions were classified as Northeast, Midwest, South, or West, following U.S. Census Bureau definitions. Urbanization-stratified analyses were limited to 1999–2020 because the NCHS 2013 Urban-Rural Classification Scheme, which relies on bridged-race population estimates, was not available in the expanded 2018–2024 CDC WONDER dataset at the time of analysis.

### 2.3. Statistical Analysis

To identify national mortality trends, Age-Adjusted Mortality Rates (AAMRs) per 100,000 population were calculated from 1999–2024 with a 95% Confidence Interval (CI). The analysis was performed after stratification of data based on variables including year, age, sex, race/ethnicity, urban–rural status, and regional variations. Age-adjusted mortality rates (AAMRs) were calculated using the direct standardization method, applying the 2000 U.S. standard population as the reference [34]. This method weights age-specific mortality rates by the proportion of the standard population in each age group, producing a summary rate that accounts for differences in age distribution across populations and time periods.

The Joinpoint Regression Program (version 6.0.1, National Cancer Institute) was used to identify statistically significant changes in mortality trends over time [35]. A maximum of 5 joinpoints was allowed, and model selection was performed using the Bayesian Information Criterion (BIC) method, which compares models starting with zero joinpoints and sequentially tests whether additional joinpoints improve model fit, selecting the most parsimonious model [36,37]. Annual Percentage Change (APC) with 95% CI was calculated using log-linear regression models applied to mortality data. APCs were classified as increasing or decreasing if the slope was significantly different from zero using two-tailed *t*-tests. A *p*-value of <0.05 was considered statistically significant [35].

## 3. Results

### 3.1. Overall Trends

A total of 241,075 deaths with respiratory failure (RF) listed among adults with chronic liver disease (CLD) were recorded in the United States between 1999 and 2024. Annual deaths increased from 3956 in 1999 to a peak of 18,728 in 2021, representing a 373.3% increase, before declining to 16,973 by 2024. The overall age-adjusted mortality rate (AAMR) rose from 2.237 per 100,000 (95% CI, 2.167–2.306) in 1999 to 7.162 per 100,000 (95% CI, 7.056–7.268) in 2021, a 3.2-fold increase, followed by a decline to 6.132 per 100,000 (95% CI, 6.037–6.228) by 2024.

Joinpoint regression analysis identified four statistically significant trend segments. From 1999 to 2006, the AAMR increased at a moderate pace (APC +2.40%; 95% CI, 1.84–2.95; *p* < 0.000001). This was followed by an accelerated increase from 2006 to 2018 (APC +5.37%; 95% CI, 5.16–5.59; *p* < 0.000001). Joinpoint regression then identified a statistically derived segment from 2018 to 2021 with a markedly accelerated increase (APC +13.10%; 95% CI, 10.52–15.75; *p* < 0.000001). It is important to note that the 2018 joinpoint was determined by the statistical model rather than by a priori clinical definition. The acceleration during this segment likely reflects both the continuation of the pre-existing upward trajectory in CLD-related mortality and the superimposed effects of the COVID-19 pandemic beginning in 2020. For transparency, this segment is referred to as the “late acceleration period (2018–2021)” throughout the manuscript, acknowledging that the pandemic’s direct effects were confined to 2020–2021. Subsequently, a statistically significant decline was observed from 2021 to 2024 (APC −4.32%; 95% CI, −5.33 to −3.30; *p* < 0.000001). Despite this post-2021 decline, the 2024 AAMR of 6.132 per 100,000 remained 174.2% above the 1999 baseline, indicating an incomplete reversal.

### 3.2. Sex-Stratified Trends

Males consistently exhibited higher AAMRs than females throughout the study period. In 1999, the AAMR was 3.084 per 100,000 (95% CI, 2.961–3.207) for males and 1.524 per 100,000 (95% CI, 1.446–1.602) for females, corresponding to a male-to-female rate ratio of 2.02. By 2021, the AAMR peaked at 8.724 per 100,000 (95% CI, 8.556–8.894) for males and 5.811 per 100,000 (95% CI, 5.679–5.945) for females, narrowing the rate ratio to 1.50. During the full study period (1999–2024), males accounted for 141,450 deaths (58.7%) and females accounted for 99,625 deaths (41.3%).

**Figure 1:**
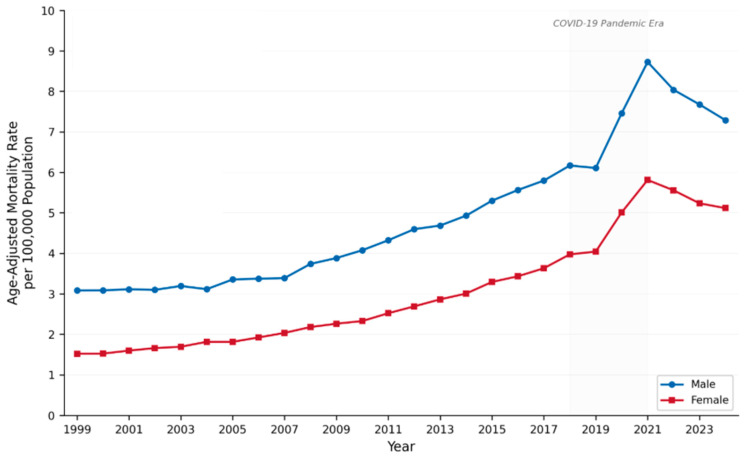
Trends in respiratory failure mortality among adults with chronic liver disease by sex, United States, 1999–2024.

Joinpoint regression revealed distinct temporal patterns by sex (Figure 1). Among females, the AAMR increased significantly across three consecutive rising segments: 1999–2006 (APC +3.52%; 95% CI, 2.67–4.37; *p* < 0.000001), 2006–2018 (APC +5.97%; 95% CI, 5.65–6.29; *p* < 0.000001), and 2018–2021 (APC +14.38%; 95% CI, 10.64–18.26; *p* < 0.000001), followed by a significant decline from 2021 to 2024 (APC −3.81%; 95% CI, −5.23 to −2.37; *p* = 0.000054). Among males, no significant change was observed from 1999 to 2004 (APC +0.07%; 95% CI, −1.36 to 1.53; *p* = 0.91), followed by a sustained increase from 2004 to 2018 (APC +4.78%; 95% CI, 4.51–5.05; *p* < 0.000001), a late acceleration period from 2018 to 2021 (APC +12.36%; 95% CI, 8.28–16.60; *p* = 0.000007), and a significant post-2021 decline (APC −4.90%; 95% CI, −6.50 to −3.28; *p* = 0.000013). Notably, the acceleration was steeper for females (+14.38%) than for males (+12.36%), though males exhibited a more rapid post-2021 decline (−4.90% vs. −3.81%).

### 3.3. Race/Ethnicity-Stratified Trends

**Figure 2:**
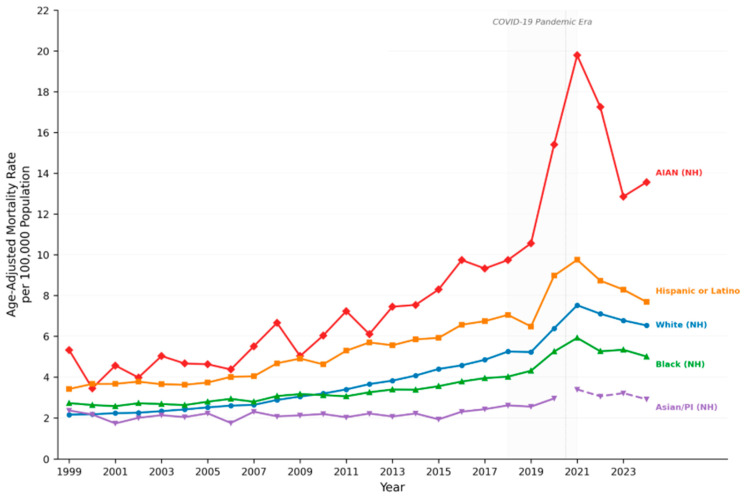
Trends in respiratory failure mortality among adults with chronic liver disease by race/ethnicity, United States, 1999–2024.

Pronounced racial and ethnic disparities in RF mortality among adults with CLD were observed throughout the study period (Figure 2). American Indian or Alaska Native (AIAN) individuals had the highest AAMRs among all groups, rising from 5.319 per 100,000 (95% CI, 4.007–6.924) in 1999 to 19.793 per 100,000 (95% CI, 18.119–21.609) in 2021, representing a 3.7-fold increase. AIAN populations experienced the most dramatic surge (APC +26.90%; 95% CI, 1.78–58.23; *p* = 0.036) during 2018–2021. The AIAN subgroup estimates should be interpreted with caution given the relatively small population size, which results in wider confidence intervals and greater year-to-year variability in mortality rates. The wide 95% CI for the 2018–2021 APC reflects this statistical instability. Nevertheless, the overall direction of the trend is consistent with prior studies documenting disproportionately high CLD mortality in AIAN populations. Following the surge, the steepest post-2021 decline of any racial/ethnic group was observed (APC −12.99%; 95% CI, −21.11 to −4.03; *p* = 0.008). The preceding period from 1999 to 2018 showed a steady increase (APC +4.91%; 95% CI, 3.66–6.17; *p* < 0.000001). Despite the post-2021 decline, the AIAN AAMR in 2024 remained markedly elevated at 13.562 per 100,000 (95% CI, 12.230–15.015).

Hispanic or Latino individuals had the second-highest AAMRs, increasing from 3.418 per 100,000 (95% CI, 3.060–3.777) in 1999 to 9.750 per 100,000 (95% CI, 9.401–10.110) in 2021. Joinpoint analysis identified a sustained rise from 1999 to 2018 (APC +4.02%; 95% CI, 3.56–4.49; *p* < 0.000001), a surge from 2018 to 2021 (APC +12.18%; 95% CI, 2.57–22.69; *p* = 0.015), and a significant post-2021 decline (APC −7.26%; 95% CI, −10.91 to −3.46; *p* = 0.001), with the 2024 AAMR declining to 7.683 per 100,000.

Among White individuals, the AAMR rose from 2.161 per 100,000 in 1999 to 7.519 per 100,000 in 2021. Four trend segments were identified: 1999–2006 (APC +2.78%; 95% CI, 2.24–3.33; *p* < 0.000001), 2006–2018 (APC +5.85%; 95% CI, 5.64–6.06; *p* < 0.000001), 2018–2021 (APC +12.98%; 95% CI, 10.47–15.53; *p* < 0.000001), and 2021–2024 (APC −3.71%; 95% CI, −4.69 to −2.73; *p* = 0.000001). The 2024 AAMR was 6.529 per 100,000.

Black or African American individuals showed a more gradual initial trajectory, with the AAMR increasing from 2.726 per 100,000 in 1999 to 5.917 per 100,000 in 2021. Four segments were identified: 1999–2011 (APC +1.66%; 95% CI, 1.01–2.31; *p* = 0.000064), 2011–2018 (APC +3.55%; 95% CI, 2.09–5.03; *p* = 0.000102), 2018–2021 (APC +13.24%; 95% CI, 5.68–21.35; *p* = 0.002), and 2021–2024 (APC −4.91%; 95% CI, −7.97 to −1.74; *p* = 0.005). The 2024 AAMR was 5.006 per 100,000.

Asian or Pacific Islander individuals had the lowest AAMRs throughout the study period and showed no significant trend from 1999 to 2015 (APC +0.06%; 95% CI, −0.79 to 0.92; *p* = 0.89), followed by a significant rise from 2015 to 2020 (APC +6.47%; 95% CI, 3.05–10.00; *p* = 0.001). For the 2021–2024 period, no statistically significant trend was detected (APC −4.03%; 95% CI, −12.34 to 5.07; *p* = 0.19). The AAMR was 2.915 per 100,000 in 2024. Among Non-Hispanic individuals, trends closely mirrored the overall pattern: 1999–2006 (APC +2.30%), 2006–2018 (APC +5.40%), 2018–2021 (APC +13.36%), and 2021–2024 (APC −4.04%), all statistically significant (*p* < 0.000001).

### 3.4. Urbanization-Stratified Trends

**Figure 3:**
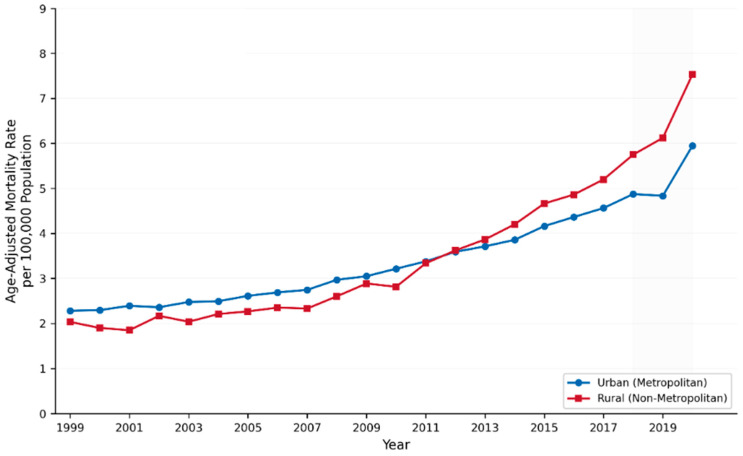
Trends in respiratory failure mortality among adults with chronic liver disease by urbanization, United States, 1999–2020. Note: Urbanization data were available only through 2020 due to the unavailability of the NCHS 2013 Urban-Rural Classification Scheme in the expanded 2018–2024 CDC WONDER dataset.

Urbanization-stratified analysis, available for 1999–2020, revealed a widening rural-urban disparity (Figure 3). At baseline in 1999, urban areas (metropolitan) had a higher AAMR (2.279 per 100,000; 95% CI, 2.201–2.357) than rural areas (non-metropolitan) (2.033 per 100,000; 95% CI, 1.875–2.191). However, by 2020, the rural AAMR (7.530 per 100,000; 95% CI, 7.242–7.819) had surpassed the urban AAMR (5.946 per 100,000; 95% CI, 5.842–6.051) by 26.6%.

Joinpoint regression identified a single joinpoint in 2007 for both strata. From 1999 to 2007, both areas showed similar moderate increases (urban: APC +2.41%; 95% CI, 0.76–4.08; *p* = 0.007; rural: APC +2.56%; 95% CI, 0.53–4.63; *p* = 0.016). After 2007, the trajectories diverged substantially: rural areas experienced a significantly steeper rise (APC +8.74%; 95% CI, 8.03–9.45; *p* < 0.000001) compared to urban areas (APC +5.51%; 95% CI, 4.94–6.10; *p* < 0.000001). Across the metropolitan subcategories (1999–2020), the highest 2020 AAMRs were observed in Large Central Metro areas, followed by Medium Metro, Small Metro, and Large Fringe Metro areas.

### 3.5. Geographic Regional Trends

**Figure 4:**
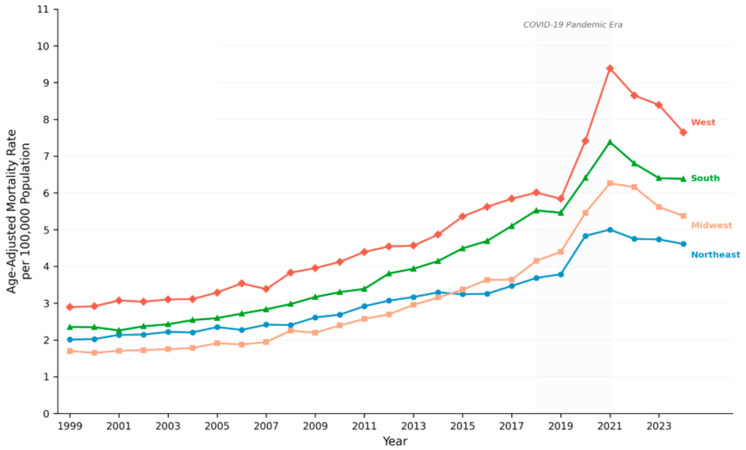
Trends in respiratory failure mortality among adults with chronic liver disease by US Census Region, United States, 1999–2024.

Among the four US Census regions, the West consistently exhibited the highest AAMRs throughout the study period, followed by the South, Northeast, and Midwest (Figure 4). In 1999, the AAMR ranged from 1.699 per 100,000 (Midwest) to 2.891 per 100,000 (West). By 2021, all regions had experienced substantial increases, with AAMRs ranging from 5.000 per 100,000 (Northeast) to 9.385 per 100,000 (West).

The Midwest experienced the steepest late-period acceleration (2018–2021: APC +16.25%; 95% CI, 9.81–23.05; *p* = 0.000047), followed by the West (APC +16.00%; 95% CI, 6.49–26.36; *p* = 0.002), the Northeast (APC +11.95%; 95% CI, 3.43–21.17; *p* = 0.008), and the South (APC +10.52%; 95% CI, 5.59–15.67; *p* = 0.0003). The pre-pandemic trajectory also varied: the Northeast showed a single sustained increase from 1999 to 2018 (APC +3.36%), while the Midwest, South, and West each demonstrated two-segment pre-pandemic trends with acceleration after 2006.

Post-2021 declines were statistically significant in the Midwest (APC −5.06%; 95% CI, −7.47 to −2.58; *p* = 0.001), South (APC −4.25%; 95% CI, −6.24 to −2.21; *p* = 0.001), and West (APC −4.68%; 95% CI, −8.22 to −1.00; *p* = 0.016), but not in the Northeast (APC −3.25%; 95% CI, −6.75 to 0.38; *p* = 0.076). By 2024, the West retained the highest AAMR at 7.644 per 100,000, followed by the South (6.379), Midwest (5.372), and Northeast (4.610).

### 3.6. Age Distribution

Age-specific analyses report crude mortality rates rather than age-adjusted rates because age adjustment is not applicable when examining mortality within a single age stratum; within each age group, the crude rate directly reflects the mortality burden without the need for standardization.

The age-specific burden of RF mortality among adults with CLD shifted over the study period. During 1999–2020, the 55–64 age group accounted for the largest proportion of deaths (51,062; 29.97%), followed by the 65–74 group (40,768; 23.93%), 45–54 group (32,870; 19.29%), 75–84 group (23,854; 14.00%), 35–44 group (10,740; 6.30%), 85+ group (8202; 4.81%), and 25–34 group (2892; 1.70%). Crude mortality rates during this period were highest in the 75–84 group (7.991 per 100,000) and the 65–74 group (7.987 per 100,000).

During 2021–2024, the 65–74 age group became the leading contributor (20,311; 28.73%), followed by the 55–64 group (19,040; 26.94%). Crude mortality rates were substantially higher across all age groups compared with the earlier period, with the 75–84 group exhibiting the highest rate (16.190 per 100,000), followed by the 85+ group (14.985 per 100,000) and the 65–74 group (14.763 per 100,000). The 25–34 group showed a 2.96-fold increase in crude rate (0.930 vs. 0.314 per 100,000), and the 35–44 group showed a 2.37-fold increase (2.732 vs. 1.153 per 100,000), indicating growing mortality across younger adults as well.

### 3.7. Place of Death

The overwhelming majority of RF deaths among adults with CLD occurred in inpatient medical facilities across both study periods. During 1999–2020 (n = 170,388), inpatient deaths accounted for 138,700 (81.4%), followed by decedent’s home (12,666; 7.4%), nursing home or long-term care facilities (7994; 4.7%), hospice facilities (5476; 3.2%), outpatient or emergency departments (2889; 1.7%), and other or unknown locations (3163; 1.9%).

During 2021–2024 (n = 70,686), the distribution of deaths remained similar, with inpatient deaths accounting for 56,627 (80.1%), followed by deaths at home (5879; 8.3%), hospice facilities (3272; 4.6%), nursing home or long-term care (2723; 3.9%), outpatient or emergency departments (1329; 1.9%), and other locations (856; 1.2%). A modest increase in the proportion of deaths occurring in hospice facilities (4.6% vs. 3.2%) and at home (8.3% vs. 7.4%) was observed in the more recent period, which may reflect changes in end-of-life care practices or a greater emphasis on palliative care referrals.

## 4. Discussion

This study provides the first comprehensive, population-level analysis of respiratory failure (RF)-related mortality among adults with CLD in the United States spanning 1999 to 2024. The findings reveal a 3.2-fold increase in the overall AAMR from 2.237 per 100,000 in 1999 to a peak of 7.162 per 100,000 in 2021, followed by a partial decline to 6.132 per 100,000 by 2024. These trends closely parallel the broader trajectory of CLD mortality in the United States, which has been driven by rising rates of ALD and NAFLD/MASLD [1,2,4,9]. The accelerated increase observed from 2006 to 2018 (APC +5.37%) aligns temporally with the documented surge in mortality from ALD- and NAFLD-related cirrhosis during this period [1,2,3,4]. The marked acceleration observed from 2018 to 2021 (APC +13.10%) is consistent with the broader acceleration of CLD mortality during the COVID-19 pandemic, attributed to both direct viral effects and indirect consequences [8,9,10,22]. The use of the broad K70–K76 ICD-10 range captures the full spectrum of CLD etiologies, consistent with the Global Burden of Disease Study methodology [38]. While some included codes (e.g., K71, toxic liver disease) may have a weaker pathophysiologic link to RF, the predominant contributors within this range—alcoholic liver disease (K70), liver fibrosis and cirrhosis (K74), and liver failure (K72)—are well-established risk factors for respiratory complications [29,39]. Future etiology-specific analyses using narrower code ranges would complement the present findings.

The acceleration in RF mortality among CLD patients during the late period (2018–2021) may reflect multiple converging factors. SARS-CoV-2 infection posed a particularly severe threat to patients with cirrhosis, with mortality rates of 32% in cirrhotic patients compared to 8% in those without cirrhosis, and respiratory failure was the leading cause of death [22,23]. The immune dysregulation inherent to advanced liver disease, including impaired innate and adaptive immunity, may have rendered these patients especially vulnerable to severe COVID-19 pneumonia and ARDS [21,24,40]. Simultaneously, the pandemic was potentially related to a substantial increase in alcohol consumption, with retail alcohol sales rising sharply and ALD hospitalizations increasing by more than 20% above pre-pandemic levels [11,12,41,42]. Modeling studies projected that even a one-year increase in alcohol consumption during the pandemic could result in 8000 additional ALD-related deaths and 18,700 cases of decompensated cirrhosis over the subsequent two decades [11]. The American College of Gastroenterology (ACG) Clinical Guideline on ALD noted that AUD-related mortality rates increased by 24.8% in 2020 and 22% in 2021 compared with pre-pandemic rates, with hospitalizations rising especially among younger individuals, women, and minorities [13]. These alcohol-driven increases in liver disease severity would be expected to potentially increase the incidence of RF through progression to decompensated cirrhosis, ACLF, and associated pulmonary complications [13,16,20]. Additionally, pandemic-related disruptions in healthcare delivery, including deferred screening, delayed specialist referrals, and reduced access to liver transplantation, may have contributed to disease progression and worse outcomes [12,40]. These interpretations are hypothesis-generating and are based on temporal associations with published literature rather than individual-level data from the present study. The ecological design precludes causal inference regarding the specific mechanisms driving the observed trends.

The post-2021 decline in RF mortality (APC −4.32%) is a positive development but warrants cautious interpretation. The 2024 AAMR of 6.132 per 100,000 remains 174.2% higher than the 1999 baseline, indicating that the decline constitutes only a partial reversal of the late-period surge rather than a return to pre-pandemic levels. This trend parallels the reduction in excess ALD hospitalizations observed at the end of 2020 and the gradual restoration of healthcare services following the acute phase of the pandemic [42]. Nevertheless, persistently elevated mortality rates indicate that structural factors such as increasing obesity, metabolic syndrome, and continued high-risk alcohol use are sustaining RF-related deaths [1,7,9]. The present study was designed as a descriptive trend analysis using joinpoint regression, which is the standard analytical approach for identifying statistically significant changes in mortality trends over time and has been widely validated in population-level mortality research [3,36,37]. The study does not aim to establish causal associations between CLD and RF, for which individual-level cohort studies with appropriate control groups and multivariable adjustment would be required.

The sex-stratified findings demonstrate that males consistently had higher AAMRs than females, consistent with the well-established male predominance in CLD mortality [3,5]. However, the narrowing of the male-to-female rate ratio from 2.02 in 1999 to 1.50 in 2021 is notable and aligns with recent evidence that CLD and cirrhosis mortality rates are increasing disproportionately in women, particularly younger women [5]. The steeper acceleration among females (APC +14.38% vs. +12.36% for males) may reflect the documented rise in alcohol use disorder among women during the pandemic [5,7,13]. Women are less likely to access AUD treatment and prevention services, potentially due to greater perceived stigma and competing family responsibilities [13]. These findings suggest that sex-specific differences in RF-CLD mortality trends warrant further investigation and may inform future public health strategies. However, the present study does not directly evaluate screening utilization or alcohol-related behaviors, and specific policy recommendations would require additional individual-level data.

Pronounced racial and ethnic disparities were observed throughout the study period. AIAN populations had the highest AAMRs and experienced the most dramatic surge (APC +26.90%), consistent with prior studies documenting disproportionately high CLD mortality in this population [3,7,8]. The ACG guideline noted that ALD-related death rates during the pandemic were highest among AIAN individuals [8,13]. These disparities likely reflect a confluence of factors, including higher prevalence of alcohol use disorder, limited access to healthcare through underfunded Indian Health Service facilities, and socioeconomic disadvantage [7,43]. Hispanic or Latino individuals had the second-highest AAMRs, consistent with prior CDC WONDER analyses showing elevated cirrhosis mortality in this population [3,43]. The relatively lower AAMRs among Asian or Pacific Islander individuals align with the known lower prevalence of ALD in this group, though hepatitis B virus-related liver disease remains a concern [2,4]. Black or African American individuals showed a more gradual initial trajectory, which may reflect the shifting etiologic landscape from viral hepatitis toward ALD and NAFLD, conditions that have disproportionately affected White and AIAN populations [6,7]. These racial and ethnic disparities in RF mortality among CLD patients mirror and amplify the broader health inequities documented in liver disease epidemiology [6,7,25].

The urbanization-stratified analysis revealed a striking reversal in the rural-urban gradient. At baseline in 1999, urban areas had higher AAMRs than rural areas, but by 2020, rural AAMRs had surpassed urban rates by 26.6%. This widening rural-urban disparity is consistent with findings from studies of cirrhosis mortality and hepatocellular carcinoma [25,43,44,45,46]. Rural populations face multiple barriers to liver disease care, including limited access to hepatologists, longer travel distances to tertiary centers, lower screening rates, and higher prevalence of metabolic risk factors such as obesity and diabetes [25,45]. Alcohol-induced death rates in rural areas are 18% to 23% higher than in urban populations [45]. The steeper post-2007 acceleration in rural RF mortality (APC +8.74% vs. +5.51% for urban areas) suggests that these structural disadvantages are compounding over time. It should be noted that urban–rural classification was assigned at the county level based on the NCHS 2013 classification scheme and may not fully capture individual-level healthcare accessibility, socioeconomic status, or residential mobility.

Geographic regional variation further highlights the heterogeneity of RF-CLD mortality across the United States. The West consistently exhibited the highest AAMRs, followed by the South, consistent with prior state-level analyses showing the highest cirrhosis mortality in states such as New Mexico, Kentucky, and Arkansas [3,25]. The Midwest experienced the steepest surge (APC +16.25%), which may reflect regional patterns of alcohol consumption and healthcare infrastructure. The Northeast had the lowest AAMRs and was the only region without a statistically significant post-2021 decline, possibly reflecting different etiologic profiles or healthcare system characteristics. County-level analyses have shown that more than 60% of the variability in liver disease mortality is explained by demographics, clinical risk factors, and access to specialty care, suggesting that targeted regional interventions could meaningfully reduce disparities [25].

The age distribution analysis revealed a shift in the peak burden from the 55 to 64 age group during 1999 to 2020 to the 65 to 74 age group during 2021 to 2024. This shift likely reflects the aging of the baby boomer cohort, which carries a high prevalence of HCV-related and alcohol-related liver disease [3]. However, the substantial increases in crude mortality rates among younger adults aged 25 to 34 (2.96-fold) and 35 to 44 (2.37-fold) are alarming and consistent with prior reports of rapidly rising cirrhosis mortality in young adults driven by ALD [3,9]. These trends suggest that RF as a terminal event in CLD is increasingly affecting younger populations, underscoring the urgency of early intervention for alcohol misuse and metabolic risk factors.

The place-of-death analysis showed that approximately 80% of RF deaths among CLD patients occurred in inpatient settings across both study periods, reflecting the acute and critical nature of RF in this population [20]. The modest shift toward increased proportions of deaths in hospice facilities (3.2% to 4.6%) and at home (7.4% to 8.3%) in the more recent period may reflect evolving end-of-life care practices and the expanding role of palliative care in advanced liver disease. These findings suggest opportunities to improve advance care planning and the integration of palliative care for patients with CLD at risk of RF. The present analysis does not exclude deaths with concurrent respiratory diagnoses such as COPD, pneumonia, or sepsis. While these comorbidities may independently contribute to RF, their co-occurrence with CLD on the death certificate reflects the clinical reality that patients with CLD frequently develop RF through multiple converging mechanisms, including CLD-specific pulmonary syndromes, immune dysregulation, and comorbid cardiopulmonary disease. Disentangling the independent contribution of each condition would require individual-level clinical data with detailed phenotyping, which is beyond the scope of death certificate-based analyses.

This study has several limitations inherent to the use of the CDC WONDER database. Death certificate data rely on the accuracy of cause-of-death coding by certifying physicians, and misclassification of both CLD and RF is possible [47,48,49]. The use of multiple-cause-of-death files captures RF listed as either an underlying or contributing cause, which may overestimate the direct causal relationship between CLD and RF. The any-mention approach is the standard methodology in multiple-cause-of-death research: a systematic review found that 87% of published multiple-cause studies used this approach [28]. Separating underlying from contributing cause would introduce its own sources of bias, as death certificate coding of underlying cause is subject to certifier judgment and automated coding algorithms that may inconsistently assign the underlying cause between RF and CLD [50]. Analyses relying solely on underlying cause of death may underestimate the disease burden captured by contributory information [51]. Future studies could examine whether trends differ when RF is designated as the underlying versus contributing cause of death. The database does not provide information on CLD etiology, disease severity (e.g., Model for End-Stage Liver Disease score or Child-Pugh class), or specific pulmonary diagnoses such as hepatopulmonary syndrome or portopulmonary hypertension, refs. [14,15] limiting the ability to identify mechanistic pathways. Additionally, the present analysis cannot distinguish RF directly caused by CLD-specific pulmonary complications from RF secondary to unrelated critical illness that coincidentally occurred in patients with CLD. Race and ethnicity data are reported by informants on death certificates and may be subject to misclassification, particularly for AIAN and Hispanic populations [49]. Urbanization data were available only through 2020, precluding assessment of pandemic-era rural-urban trends. As an ecological study using aggregated death certificate data, the present analysis cannot adjust for individual-level confounders such as comorbid respiratory or cardiovascular diseases, disease severity, or specific CLD etiology. Individual-level data on alcohol consumption, smoking status, and other behavioral risk factors are not recorded on U.S. death certificates and therefore cannot be incorporated into CDC WONDER analyses. The temporal associations between observed mortality trends and published data on alcohol consumption patterns during the pandemic are hypothesis-generating and presented as such, not as causal conclusions. Multiple subgroup analyses were performed without formal correction for multiple comparisons, which increases the possibility of chance findings in certain subgroup comparisons. However, this approach is consistent with the exploratory, descriptive nature of the study and with prior CDC WONDER trend analyses, which similarly did not apply multiplicity corrections [3,5,8,30].

Despite these limitations, this study has notable strengths. It represents the first population-level analysis of RF mortality specifically among adults with CLD, spanning a 26-year period and encompassing more than 241,000 deaths. The use of joinpoint regression analysis allowed the identification of statistically significant temporal inflection points, providing granular insight into the dynamics of mortality trends. Stratification by sex, race and ethnicity, urbanization, geographic region, age, and place of death offers a comprehensive demographic and geographic characterization of this mortality burden.

## 5. Conclusions

RF-related mortality among adults with CLD in the United States increased more than threefold from 1999 to 2021, with a dramatic acceleration followed by a partial but incomplete decline. Persistent disparities by sex, race and ethnicity, urbanization, and geographic region highlight the need for targeted public health interventions. The observed disparities provide an evidence base for future studies evaluating expanded screening and treatment for ALD and MASLD, improved access to hepatology and critical care services in rural and underserved communities, and culturally tailored interventions for disproportionately affected populations. While specific policy recommendations are beyond the scope of this descriptive analysis, the persistent and widening disparities identified herein underscore the urgency of addressing structural determinants of CLD-related mortality. Future research should examine etiology-specific contributions to RF mortality in CLD and evaluate the impact of emerging therapies on long-term outcomes.

## Data Availability

Data are publicly available through CDC WONDER (https://wonder.cdc.gov, accessed on 3 July 2026).
